# Therapeutic Targeting of the *Staphylococcus aureus* Accessory Gene Regulator (*agr*) System

**DOI:** 10.3389/fmicb.2018.00055

**Published:** 2018-01-25

**Authors:** Li Tan, Si Rui Li, Bei Jiang, Xiao Mei Hu, Shu Li

**Affiliations:** ^1^Department of Microbiology, College of Basic Medical Sciences, Third Military Medical University, Chongqing, China

**Keywords:** *Staphylococcus aureus*, *agr*, virulence factors, biofilms, autoinduction

## Abstract

*Staphylococcus aureus* can cause numerous different diseases, which has been attributed to its large repertoire of virulence factors, many of which are under the control of the accessory gene regulator (*agr*) quorum sensing system. Under conditions of high cell density, *agr* increases the production of many virulence factors, decreases expression of several colonization factors, and is intimately associated with the pathogenesis and biofilm formation of *S. aureus*. This review summarizes our current understanding of the molecular mechanisms underlying *agr* quorum sensing and the regulation of *agr* expression. The discussion also examines subgroups of *agr* and their association with different diseases, and concludes with an analysis of strategies for designing drugs and vaccines that target *agr* to combat *S. aureus* infections.

## Overview of Quorum Sensing and *agr* in *Staphylococcus aureus*

Quorum sensing is a bacterial cell to cell communication system that controls expression of many genes in response to population density (Fuqua et al., 1994). The phenomenon was first investigated in the marine bacterium *Vibrio fischeri*, in which it modulates the expression of bioluminescence (Engebrecht et al., 1983). Subsequently, quorum-sensing systems have been found in a wide variety of microbes, and the main similarities and differences in the mechanisms employed by Gram-positive and Gram-negative bacteria have been describe (Xavier and Bassler, 2003). Gram-negative bacteria primarily use the LuxI/LuxR system, in which homoserine lactone (HSL) autoinducers are synthesized by LuxI-type enzymes and detected by LuxR-type transcriptional regulators. Gram-positive bacteria typically use oligopeptide-mediated quorum sensing, and two-component sensor kinase phosphorylation cascades are employed for signal transmission (Bassler, 2002).

*Staphylococcus aureus* is a highly versatile and adaptable Gram-positive pathogen. It can inhabit the skin and mucous membranes as a harmless commensal (Novick, 2003). However, *S. aureus* can also proliferate in the bloodstream and in various tissues, causing serious disease (Krismer and Peschel, 2011), and is considered one of the leading causes of hospital- and community-acquired infections worldwide (Mandal et al., 2015). It can cause conditions ranging from minor skin infections to systemic, life-threatening illnesses, such as pneumonia, osteomyelitis, and endocarditis (Thammavongsa et al., 2015). A significant aspect of diseases caused by *S. aureus* is recurrence, which is seen in 8–33% of skin, soft-tissue, and bloodstream infections, resulting in severe human morbidity and mortality (Thammavongsa et al., 2015).

The ability of *S. aureus* to cause such a wide range of infections is attributed to its large arsenal of virulence factors (adhesins, toxins, and enzymes) (Tuchscherr and Loffler, 2016), many of which are under the control of the quorum-sensing accessory gene regulator (*agr*) system (Li et al., 2014). The *agr* locus was first described by Peng et al. (1988) and found to be widespread in staphylococci. The *agr* system serves a crucial role in pathogenesis by regulating virulence factors, biofilm formation, and the heterogeneous resistance of methicillin-resistant *Staphylococcus aureus* (MRSA) (Singh and Ray, 2014; Mohsenzadeh et al., 2015; Kavanaugh and Horswill, 2016).

**FIGURE 1:**
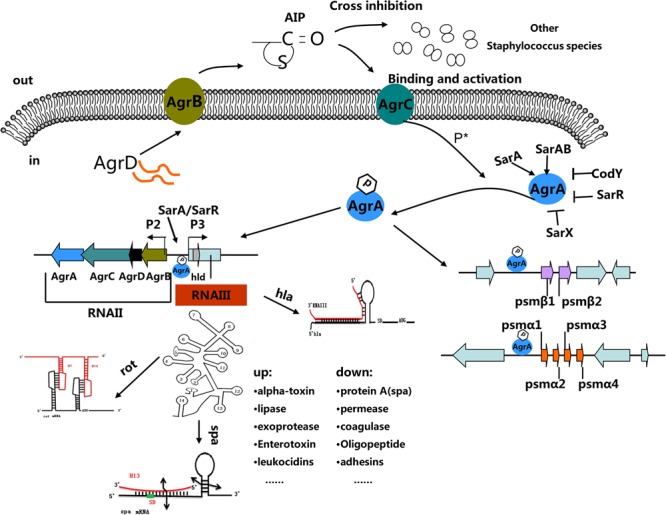
The *Staphylococcal* quorum-sensing system. The *agr* locus is composed of divergent transcripts designated RNAII and RNAIII, driven by promoters P2 and P3, respectively. The AIP signal is produced from the AgrD precursor, while the membrane-localized enzyme AgrB participates in the maturation and export of the AIP. At a critical threshold concentration, AIP activates the two-component signal transduction system, AgrC–AgrA, and causes the phosphorylation of AgrA. Once phosphorylated, AgrA binds to the P2 and P3 promoter regions, as well as promoters PSM-α and PSM-β, resulting in *agr* system transcription. RNAIII encodes the delta-toxin encoding gene *hld*, and 14 stem-loop motifs. These domains regulate the expression of numerous virulence factors. Other regulators (such as SarA, SrrAB, SarR and SarX) can enhance or inhibit *agr* activity.

The *agr* operon is organized around two divergent promoters, P2 and P3, and generates two primary transcripts, RNAII and RNAIII, respectively (**Figure 1**) (Ji et al., 1995). RNAII encodes AgrB, AgrD, AgrC, and AgrA. AgrD encodes the precursor of the autoinducing peptide (AIP) pheromone. AgrB is a multifunctional endopeptidase and chaperone protein that contributes to the maturation and export of AIP. AgrC and AgrA comprise a two-component signal transduction system in which AgrC is the membrane histidine kinase and AgrA is the response regulator (Novick et al., 1995). The *agr* system is activated when the extracellular AIP concentration reaches a threshold. Upon binding AIP, AgrC phosphorylates AgrA, which in turn activates the P2 and P3 promoters in addition to several other transcriptional targets (Ji et al., 1995; Queck et al., 2008). RNAIII is a posttranscriptional regulator of multiple virulence genes. Recognizable *agr* loci are subject to considerable sequence polymorphism. After cloning and initial characterization of the *agr* locus, Peng et al. (1988) identified four variants (*agr* types I through IV). These *S. aureus* strains are characterized by mutations in the sensor domain of the histidine kinase AgrC and polymorphisms in the sequences of secreted autoinducing peptides (Srivastava et al., 2014), affecting the three determinants of *agr* group specificity (AgrB, AgrD, and the sensor domain of AgrC) (**Figure 1**) (Wright et al., 2005b). Because *agr* is an integrated system, these variations must evolve in concert in order to maintain *agr* functionality which enable the bacteria to evade host defenses, spread within the host, and to degrade host cells and tissues (Kavanaugh and Horswill, 2016).

## Molecular Basis of the *agr* System in *S. aureus*

AgrB, the 22 kDa peptidase responsible for proteolysis of AgrD, is located in the cytoplasmic membrane. It has six transmembrane segments, consisting of four hydrophobic transmembrane α-helices and two hydrophilic loops with several positively charged amino acid residues (Zhang et al., 2002). AgrB is the most unusual feature of the staphylococcal *agr* system because its sequence has little in common with other quorum-sensing proteins. In staphylococcal species, the N-terminal domain of AgrB is highly conserved, the first 34 residues, located in the first transmembrane hydrophilic domain, are absolutely conserved among the four *S. aureus agr* types (Thoendel et al., 2011). Mutations in this conserved region will eliminate AgrB activity (Qiu et al., 2005). In particular, the histidine residue at position 77 (H77) and the cysteine residue at position 84 (C84) are required for the proteolytic processing of AgrD. Mutations in the second hydrophilic transmembrane domain have no effect on AgrB activity. All AgrB homologs are likely to utilize the same or similar mechanisms to process AgrD, but the mechanism of AgrD-dependent AIP maturation and the AIP secretion pathway are unknown. AgrB and AgrD are sufficient for AIP production, since heterologous expression of AgrB and D in *Escherichia coli* or *Bacillus subtilis* results in functional AIP (Thoendel et al., 2011). Chimeric AgrB proteins have been used to identify the group-specific segment(s) in AgrB that contribute to AgrD proteolysis. The results indicate that the interaction between AgrB and AgrD is group specific (Zhang and Ji, 2004). For example, in *agr* group I, the first transmembrane α-helix and the extracellular loop 1 of AgrB are critical for processing group I AgrD. In contrast, two hydrophilic parts of group II AgrB play a key role in the processing of group II AgrD (Zhang and Ji, 2004).

AgrD is the propeptide for AIP. The structure of AgrD has diverged across species, but is typically comprised of an N-terminal amphipathic leader, a middle region of seven to nine residues that is processed into the mature AIP thiolactone structure, and a negatively charged C-terminal recognition sequence (Ji et al., 1995). AgrB-catalyzed proteolysis cuts off the AgrD recognition sequence as a linear peptide and subsequently processes it into a thiolactone group at the C-terminus of the remaining fragment. The recognition sequence of staphylococcal AgrD has abundant acidic residues and is highly conserved (Thoendel and Horswill, 2009). The AgrD propeptide is integrated in the cytoplasmic membrane by a conserved amphipathic α-helical motif in its N-terminal region that is required for the stabilization of AgrD and the production of mature AIP. However, this region is not specifically involved in the interaction with AgrB (Thoendel et al., 2011). If the N-terminal amphipathic motif of AgrD is replaced by an artificial amphipathic peptide, production of AIP still occurs (Zhang et al., 2004). Evidence suggests that the conserved cysteine residue is required to generate the thiolactone ring structure of AIP. The C-terminal tail of AgrD plays an essential role in cleavage by AgrB and AIP production (Thoendel and Horswill, 2009). It is assumed that the highly conserved Glu-Asp pair of AIP is crucial for processing the C-terminal end of the AIP (Dufour et al., 2002). The first nine residues of AgrD are necessary for AIP production and AgrB endopeptidase activity. Mutations affecting glutamate 34 or leucine 41 inhibit AIP production and AgrB activity (Thoendel and Horswill, 2009). AgrD function has been extensively studied (Thoendel and Horswill, 2009; Schwartz et al., 2014). Schwartz et al. (2014) demonstrated that AgrD structure and function are similar to the PSM family of toxins. Similar to PSMs, N-AgrD is present in the amyloid fibrils of *S. aureus* biofilms, and can form and seed amyloid fibrils *in vitro* (Schwartz et al., 2014). An AgrD mutant displays significantly reduced biofilm formation in *Listeria monocytogenes* (Riedel et al., 2009).

AgrC, the critical receptor protein for signal recognition and transmission (George Cisar et al., 2009), is a 46 KDa membrane protein belonging to the class 10 receptor-histidine protein kinase (HPK) family. Its features include an N-terminal, membrane-integrated sensor module that detects and binds AIP, several transmembrane domains, and a C-terminal histidine kinase module (Grebe and Stock, 1999).

After AIP binds to the AgrC N-terminal sensor module, a conformational change occurs in the AgrC cytoplasmic helix that links the sensor and kinase domains, which then enables autophosphorylation and activation of the AgrC kinase. The AgrC–AIP and AgrC–AgrA interaction have been intensively studied. Geisinger et al. (2009) first reported constitutive mutants of AgrC and elucidated the mechanism of ligand-receptor interaction. Additionally the team found that substitution of amino acid isoleucine at position 171 led to altered activity of AgrC (Geisinger et al., 2009). Mairpady Shambat et al. (2016) found that substitution of tyrosine by cysteine at position 223 (Y233C) in AgrC destabilizes AgrC–AgrA interaction, affecting the regulation of virulence genes that switch the strain from a cytotoxin-mediated phenotype to a colonizing phenotype. The same laboratory used random mutagenesis to isolate AgrC mutants with constitutive activity, as well as those with altered specificity for divergent AIPs. Even changes at a single amino acid affect virulence properties and infection outcome (Mairpady Shambat et al., 2016).

AgrA, the 27 kDa response regulator for the *agr* system, belongs to a family of conserved response regulators with CheY-like receiver domains (Traber and Novick, 2006). Sequence comparison indicates that the amino acid sequences of AgrB, AgrC, and AgrD are strikingly variable among different *S. aureus agr* types, whereas AgrA is highly conserved. AgrA acts as a response regulator by binding to recognition sites in RNAIII and RNAII promoter domains (Koenig et al., 2004). Using electrophoretic mobility shift assays (EMSAs), Koenig et al. (2004) demonstrated that AgrA binds to the P2-P3 region of the *agr* locus with high affinity, and the affinity of phosphorylated AgrA is stronger for the P2 promoter than for P3. However, the frameshift mutation produced by inserting an extra adenine into the seven continuous adenines in the C-terminus of AgrA generates a partially defective AgrA that significantly delays the activation of the *agr* locus (Traber and Novick, 2006). In DNA microarray experiments, Queck et al. (2008) demonstrated that AgrA up-regulates three other chromosomal operons (*psmα, psmβ*, and MW00370/0372). The up-regulations of α and β PSM transcriptions are induced by direct binding of AgrA to their promoter domains, independently of RNAIII (Thoendel et al., 2011).

## RNAIII

The 514 nucleotides of RNAIII contain 14 potential stem-loop structures. Regions in the folded molecule participate in two long-distance interactions (**Figure 1**) (Bronesky et al., 2016). The 3′-end of RNAIII which contains some C-rich sequence motifs and unpaired regions that contribute to the initiation of the binding of RNAIII to the ribosome binding sites of several target mRNAs (Bronesky et al., 2016). The 3′-end domain of RNAIII represses the synthesis of several surface and secreted proteins specific to *S. aureus* (**Figure 1**). RNAIII was the first example as an “antisense RNA” that stimulates translation of its target mRNA (Morfeldt et al., 1995). Following this discovery, many studies have investigated how RNAIII functions as an effector molecule in the *agr* system. In RNAIII mutants, low molecular weight toxins and the exoenzymes (Ecp protease and Geh lipase) are down-regulated (Xiong et al., 2002).

## Aip Synthesis, Structure and Activity

The *agr* autoinducing peptide (AIP) varies from 7–9 amino acids in length and contains a 5-membered ring. For *S. aureus*, the AIP sequences of *agr*-I, II, III, and IV are YSTCDFTM, GVNACSSLF, YINCDFLL, and YSTCYFTM, respectively (Yarwood and Schlievert, 2003). *S. aureus* must produce sufficient amounts of thiolactone-containing AIP to enable quorum sensing. The proteolytic events and chemical steps that enable AIP production have been identified *in vitro* by reconstituting the AgrB-dependent proteolysis of the AgrD precursor (Wang et al., 2015). After removal of the C-terminal tail, the new C terminus forms a thiolactone bond by condensation of the sulfhydryl group of a conserved cys residue and the α-carboxyl group. Cleavage of the N-terminal domain then results in a molecule with a tail of 2–4 amino acid residues connected to a 16-membered macrocycle (Geisinger et al., 2009). Efficient thiolactone production is driven by association of the thiolactone-containing intermediate with the membrane, which stabilizes the macrocycle, and by rapid degradation of the C-terminal fragment of AgrD after proteolysis (Wang et al., 2015).

The four AIP molecules are sufficiently similar in structure that they can bind to the AgrC receptor from different group, although in such cases they do not activate the AgrA protein inside the cell (Jabbari et al., 2012). MDowell et al. (2001) found that synthetic group I AIP analogs can replace the authentic group I AIP to activate the *agr* system. This indicates that covalent modification of the AgrC receptor is not a necessary prerequisite for *agr* activation (MDowell et al., 2001). The C-terminal endocyclic amino acid residue (aspartate) and the central cysteine are critical for the function of *S. aureus* group I AIP. Replacement of them with alanine converts the AIP from an activator to a potent inhibitor (MDowell et al., 2001).

Autoinducing peptide interactions between different *agr* groups can result in cross-inhibition, leading to quorum sensing interference (Ji et al., 1997). Structure-activity analyses on AIP–AgrC interaction indicated that AIP macrocycle size and conformation are essential to its specific activity. Johnson et al. (2015) investigated that alterations of microcycle size and conformation of AIP drastically affected its ability to bind and activate the AgrC-I receptor (Johnson et al., 2015). Swapping the five divergent residues in the second extracellular loop of the AgrC-I and AgrC-IV receptors switches the activation specificity between AIP-I and AIP-IV (Geisinger et al., 2008). These results suggest that the inhibitory receptor conformation stabilized by non-cognate AIPs is critical for the ligand–receptor interaction (Johnson et al., 2015).

## The Biological Activities of *agr* System-Mediated Regulation

*Agr* has various biological functions. The typical two are regulating the expression of staphylococcal virulence factors and facilitating the structuring and detachment of bacteria biofilms. These functions are crucial for the pathogenesis of staphylococci and are always associated with the pathogenicity of highly virulent *S. aureus*.

### *Agr* Regulation of Staphylococcal Virulence

The *agr* system is a global regulator of staphylococci and exhibits a dual regulatory effect on staphylococcal virulence (Arvidson and Tegmark, 2001; Bronner et al., 2004; Singh and Ray, 2014). It can up-regulate the expressions of several exoproteins (e.g., α-, β-, γ-hemolysin, and leucotoxins), lipases, phenol-soluble modulins, and toxic shock syndrome toxins (TSST), and represses the transcription of some cell wall-associated proteins (e.g., protein A, coagulase, and fibronectin binding protein) (Bronner et al., 2004). *Agr* can regulate the expression of virulence factors directly and indirectly. For example, through direct binding of AgrA to PSM promoter regions, the *agr* system regulates the expression of PSM (Peschel and Otto, 2013). Also, the *agr* system controls the expression of genes encoding alpha-hemolysin (*hla*), beta-hemolysin (*hlb*), protein A (*spa*), exfoliative toxin A (*etaA*), toxic shock syndrome toxin-1 (*tsst*), and staphylococcal serine protease (*sspA*) by regulating RNAIII. Through direct base pairing with target gene cohorts, or indirect control of regulating transcriptional regulators such as Rot, SarT, and SarS, RNAIII up- or down-regulate virulence gene expression (**Figure 1**) (Arvidson and Tegmark, 2001; Le and Otto, 2015; Bronesky et al., 2016).

## *Agr*-Mediated Biofilm Formation

*Staphylococcus aureus* is a leading cause of chronic relapsing infections such as implanted device related infections: intravenous catheters, urinary catheters, and orthopedic prosthesis (Singh and Ray, 2014). These types of infections all have a biofilm component which physically protects the bacteria from the immune system and cells within a biofilm are more tolerant to antibiotics (Waters et al., 2016). In *Staphylococcus*, the *agr* system appears to influence biofilm formation at structuring and dispersal stages. Many researches demonstrated that repression of *agr* is necessary for biofilm formation, while activation of the *agr* system is essential for the detachment of biofilm (Vuong et al., 2000; Boles and Horswill, 2008). Some dysfunctional *agr* mutants have been isolated from biofilm-associated infections and these form thicker, smoother biofilms (Yarwood and Schlievert, 2003).

The *agr* system can affect biofilms development in a variety of ways. In established biofilms, adding AIP could reactivate of *agr* and contribute to biofilm detachment by increasing secretion of extracellular proteases (Boles and Horswill, 2008). Solano et al. (2014) also showed that *agr* system influence biofilm development by interfering with protease expression. AgrB is also thought to regulate biofilm dispersal, because biofilm biomass (cells, extracellular polymeric substances, and extracellular DNA) is inversely correlated with *agrB* expression (Grande et al., 2014). The *agrD* mutant formed larger biofilms than did the parent strain in a static biofilm system (Yarwood et al., 2004). RNAIII controls both biofilm formation and accumulation (Coelho et al., 2008), and high RNAIII is thought to have anti-biofilm effects (Lauderdale et al., 2009). Moreover, AgrA-controlled PSM expression is also involved in biofilms detachement (Dastgheyb et al., 2015). As monomers, PSMs promote biofilm disassembly, but when polymerized in amyloid-like fibers, they favor biofilm development (Solano et al., 2014).

## Regulation of the *agr* System

As a global regulator, the *agr* system controls the expression of numerous effectors. However, its activity is under the strict control of other regulators. In addition to the autoregulatory behavior of AgrA, which binds to the P2–P3 promoter region and regulates P2 and P3 transcriptions, other factors controlling *agr* expression have been described (Reyes et al., 2011). For example, the P2–P3 intergenic region contains SarA/SarR binding sites as well as the four AgrA boxes to which AgrA binds (**Figure 1**) (Reyes et al., 2011). It was reported that SarA activates whereas SarR represses P2 transcription (Reyes et al., 2011). Two-component system SrrAB can also affect the activity of *agr* system (Pragman et al., 2004). The global regulator CodY indirectly represses *agr* activity to prevent inappropriate *agr* expression at low cell densities (Painter et al., 2014). A lack of SigB activity leads to increased RNAIII expression, thus elevating extracellular protease levels and influencing the murein hydrolase activity (Lauderdale et al., 2009). Moreover, numerous environmental and metabolic factors such as pH, glucose concentration, reactive oxygen species (ROS), and nutrient availability, can also modulate *agr* quorum sensing system in *S. aureus* (James et al., 2013).

## Association of *agr* Types With Specific Biological Characteristics

*agr* groups vary by clonal lineages distribution, antibiotic resistance profile, biofilm formation, expression of virulence factors, and AIP structures. Many studies have attempted to associate *agr* types with one or more of these characteristics (Gomes et al., 2005; Ikonomidis et al., 2009; Nichol et al., 2011).

### *Agr* Types and Clonal Lineages

Specific *S. aureus* lineages may correlate with different *Agr* types. *Agr* group I is usually found in clonal lineages CC8, CC25, CC22, CC45, and CC395. CC5, CC12, and CC15 isolates usually harbor *agr* group II, CC30 is often characterized by *agr* group III, and CC121 harbors *agr* group IV (Holtfreter et al., 2007).

### Biofilm Formation among *agr* Types

The association between *agr* groups and biofilm formation has been widely studied. Strains of *agr* groups II and III are the main biofilm producers among the four *agr* types. Ikonomidis et al. (2009) found that *agr* group II MRSAs exhibit higher biofilm formation capacity compared with the other *agr* groups. Cafiso et al. (2007) also reported that *agr* group II *S. aureus* strains are usually prolific biofilm formers, while strains in *agr* group III are less so. However, Khoramrooz et al. (2016) reported a significant association between *agr* group III and biofilm production in *S. aureus* isolates, and concluded that the type III isolates are potent biofilm producers. The relationship between *S. aureus agr* groups and antibiotic resistance is also of interest. For example, *agr* group I is more strongly associated with CA-MRSA genotypes, while *agr* group II is more correlated with HA-MRSA in human isolates (Nichol et al., 2011). In addition, another study reported that methicillin resistance of bovine isolates is more prevalent in *agr* group I than other groups (Mohsenzadeh et al., 2015).

### Toxin Gene Distribution among *agr* Types

According to an analysis performed by Jarraud et al. (2002) on 198 *S. aureus* strains, toxin gene distribution is strongly related to *agr* phylogeny, as determined using AFLP clusters. The enterotoxin gene cluster (*seg, sei, sem, sen*, and *seo*) was relevant to group IV, and correlated negatively with *agr* groups I and II. *lukD-lukE* and *hlg-2* correlated negatively with group III but were associated with other groups. Meanwhile, *eta* and *etb* were related to group IV (Jarraud et al., 2002). de Almeida et al. (2013) also evaluated the association of genes encoding cytotoxin, adhesins, and toxins with superantigen activity with *S. aureus* clones isolated from milk obtained from ewes exhibiting clinical and subclinical mastitis. The *clfA* gene was identified in all isolates, and *hla* and *lukE-D* genes were, respectively, detected from 77.3 and 82.8% clones. In contrast, *bbp, ebpS, cna, fnbB, icaA, icaD, bap, hlg, lukM-lukF-PV*, and *se-a-b-d-e* were not found (de Almeida et al., 2013).

Mobile genetic elements (MGEs) may also show *agr* group specificity. Staphylococcal chromosomal cassette *mec* (SCC*mec*) carries the *mecA* gene which encodes a penicillin-binding protein (PBP2a) and confers resistance to β-lactam antibiotics. Eleven distinct SCC*mec* elements have been identified in MRSA (Wright et al., 2005b). Interestingly, all are found in *agr* groups I, II, and III, but group IV strains have not acquired a SCC*mec* element (Wright et al., 2005b). Plasmids and phages, two common types of mobile genetic elements, are also *agr* type-specific. For example, phages and plasmids make frequent appearances in *agr*-IV strains that carry *eta* or *etb. Agr*-II strains harbor *cna* showed much lower frequencies than other types, while the TSST-1 prototype antigen is preferentially carried by *agr*-III strains (Wright et al., 2005b). Because MLST patterns occur within a single *agr* group, this suggests that *agr* groups evolved prior to MLST diversification (Wright et al., 2005b).

## *Agr* Types and Disease

Several studies have demonstrated a strong relationship between *agr* types and particular diseases. Jarraud et al. (2002) found that phylogenetic group AF1 (*agr* group IV) strains are closely related to generalized exfoliative syndromes and bullous impetigo. Among suppurative infections, endocarditis is mainly caused by phylogenetic group AF2 (*agr* groups I and II) strains. *Agr* group III and IV strains are associated with TSST-1 (Gomes et al., 2005). Sakoulas et al. (2003) determined that more than half of clinical MRSA bloodstream isolates belong to *agr* group II. Although the precise relationship is unclear, the limited literature suggests a link between different *agr* types and certain staphylococcal syndromes. The inconsistencies in these reports may reflect ecological and geographical factors or different experimental designs, but the general lessons learned from them are comparable.

## Future Perspective

Due to its importance in regulating virulence factor production and biofilm formation, the *agr* system is considered as an attractive therapeutic target. Interfering with the *agr* system or blocking it entirely may be an effective method for weakening the virulence of staphylococcal pathogens and controlling staphylococcal disease. Measures that target AgrB/D/C/A, AIP, or RNAIII are all of interest (**Table 1**).

**Table 1:** Known targets in *agr* system (target AgrB/D/C/A, RNAIII, and AIP) with the potential to inhibit *Staphylococcus aureus* infections.

Anti-*agr* compound	Mechanism of inhibition	Reference
**RNAIII inhibiting protein**		
RIP	Inhibits synthesis of *agr* transcripts RNAII and RNAIII	Gov et al., 2001
RIP derivatives (16P-AC)	Inhibits the expression of biofilm-related genes in *S. aureus*	Zhou et al., 2016
RIP-V, RIP-L	Down-regulates RNAIII expression and α-hemolysin production	Ma et al., 2015
**AIP and AIP derivatives**		
Truncated AIP-I, II, III,	Inhibits autoinduction of all four *S. aureus* subgroups	Otto et al., 2001
Vaccination with hapten-linked AIP IV	Provides passive immunity and reduces the pathology of *agr* IV strains	Tal-Gan et al., 2016
**Secondary metabolites**		
Solonamide/Solonamide B	From marine bacteria; functions via competitive inhibition of AgrC	Wang and Muir, 2016
Cochinmicin	From actinomycetes, functions via competitive inhibition of AgrC	Wang and Muir, 2016
Avellanin	From sponges; functions via competitive inhibition of AgrC	Wang and Muir, 2016
3-oxo-C12-HSL, (HQNO)	From *Pseudomonas aeruginosa*; quenches *S. aureus* autoinduction	Wang and Muir, 2016
Naringenin	Reduces *agrA* and *hla* transcript levels	Zhang et al., 2013
2-(4-methylphenyl)-1,3- thiazole-4-carboxylic acid, 9H-xanthene-9-carboxylic acid, 4-phenoxyphenol	Binds C terminus of AgrA and disrupts AgrA-DNA binding activity	Leonard et al., 2012
Savirin	Blocks *S. aureus* autoinduction	Sully et al., 2014
ω-hydroxyemodin (OHM)	Prevents *agr* activity by all four *S. aureus agr* group strains	Daly et al., 2015
**Antisense oligonucleotides**		
PLNA34	Specifically and significantly reduces *agrA* mRNA levels	Da et al., 2017
**Bacterial**		
*Staphylococcus schleiferi*	Functions by cross-inhibition of the pathogenic *agr* system	Canovas et al., 2016

### AgrB/D/C/A As *agr* System Targets

As noted earlier, the *agr* operon is transcribed from divergent promoters, P2 and P3, to yield RNAII and RNAIII, respectively. Transcription depends on the specific binding of activated AgrA to the P2 and P3 promoter regions. Because activated AgrA itself is the final product of RNAII activation, RNAII transcription can be affected by targeting AgrB, D, C, or A, as well as AIP.

Since it has a key role in *agr* activation, AgrA can serve as a potential drug target for inhibition of *agr* quorum sensing. Blocking the binding of phosphorylated AgrA to the P2 and P3 promoters will repress the *agr* system, interfering with the expression of virulence factors such as α-hemolysin and PSMs, and attenuating the virulence of *S. aureus* (Khodaverdian et al., 2013). Pathogens affected in this way are less able to colonize host tissues and are ultimately eradicated by host immune system (Kong et al., 2016). Considerable effort has focused on identifying AgrA antagonists. Leonard et al. (2012) recently identified three compounds that target the LytTR DNA binding domain of AgrA and prevent its binding to the P3 promoter. Savirin, a small molecule that inhibits activation of P3 by AgrA in all four *agr* types, was also screened by Sully et al. (2014). This compound prevents the up-regulation of virulence genes in *S. aureus*, promotes clearance of *agr*+ *S. aureus*, and has a high efficacy in murine skin infection models (Sully et al., 2014). ω-hydroxyemodin (OHM), a polyhydroxyanthraquinone isolated from solid-phase cultures of penicillium restrictum, prevents interaction of AgrA with the P2 promoter, thus blocking *agr* activity of all *S. aureus agr* types (Daly et al., 2015). Naringenin significantly reduces AgrA and *hla* transcript levels in post-exponential cultures and protects mice from pneumonia caused by *S. aureus* (Zhang et al., 2013). Solonamide B cyclodepsipeptide isolated from the marine bacterium *Photobacterium halotolerans*, strongly down-regulates the expression of RNAIII and AgrA-controlled virulence genes in *S aureus*. Moreover, because the phosphorylation and activation of AgrA is catalyzed by AgrC, inhibitors targeting AgrC or AgrA are also potential ways to block disease development. For example, cochinmicin, avellanin, and Solonamide possess a 16-membered macrocycle and can function as competitive inhibitors of AgrC (Wang and Muir, 2016). Another approach for quenching the *agr* system is to use antisense locked nucleic acids that target the members of *agr* system. The antisense oligonucleotide PLNA34, designed to target *agrA* mRNA, specifically and significantly down-regulated *agrA* mRNA transcription with no bactericidal activity (Da et al., 2017).

AgrB and AgrD are responsible for producing autoinducing peptide (AIP), and inhibitors that target AgrB or prevent export of AgrD would be efficient antagonists of the *agr* system. Examples include peptide analogs that irreversibly antagonize the cleavage and cyclization of the AgrD active site, destabilize the enzyme structure of AgrB, or transform it to an inactive conformation (Gray et al., 2013).

### *Agr* Targeting by Cross-Inhibitory AIP

Some secondary metabolites have the potential to interfere with bacterial signals (Packiavathy et al., 2014; Zhang et al., 2014), because they can serve as both autoinducer agonists and antagonists. Some AIPs from other staphylococcal species are able to repress the function of the *S. aureus agr* system. It may be possible to use the *agr* pheromone from a non-pathogenic strain of *S. aureus* or from other staphylococci for therapeutic purposes against *S. aureus* infection (Jabbari et al., 2012). For example, the culture supernatant of *S. schleiferi* exhibits potent inhibitory activity against the *S. aureus agr* system and is effective against all four *agr* classes. The expression of many virulence genes is also suppressed when *S. aureus* and *S. schleiferi* are co-inoculated *in vivo* (Canovas et al., 2016). Genes that contribute to colonization and virulence regulated by the *agr* system are also inhibited by coculture with other commensal strains (Ramsey et al., 2016). Furthermore, AIP can be inhibited by other AIP subtypes of *S. aureus* and analogs from staphylococci. AIP-II has been applied to prevent abscess formation caused by *agr*-I *S. aureus* in a murine model (Wright et al., 2005a). The *agr* system of *S. aureus* and *S. epidermidis* are also cross-inhibitory with each other. The AIPs of *S. aureus* groups I, II, and III are all sensitive to *S. epidermidis* pheromone, while subgroup 4 pheromones of *S. aureus* can also inhibit the *S. epidermidis agr* response (Otto et al., 2001). AIP-I, -II, -III, and their analogs have been successfully made into quorum sensing blocking agents capable of inhibiting the autoinduction of the four *S. aureus* subgroups (Otto et al., 2001; Tal-Gan et al., 2016).

### RNAIII as a Target

As the main effector molecule of the *agr* system, RNAIII is responsible for the expression of a huge number of virulence genes. Inhibiting RNAIII may therefore be an effective method for reducing the production of toxins and other virulence factors. The RNA-III inhibiting peptide (RIP) effectively suppresses diseases caused by *S. aureus* (Gov et al., 2001). Native RIP (YSPWTNF-NH2) and some of its synthetic analogs were found to inhibit RNAII and RNAIII transcription (Gov et al., 2001). Furthermore, RIPs reduce bacterial adherence to mammalian cells and plastic substrates, and prevent biofilm formation in surgical transplantation by inhibiting RAP (Gov et al., 2001).

RNA-III inhibiting peptide derivatives have also been designed and evaluated for their potential as specific drug candidates for treating *S. aureus* infections. Ma et al. (2015) reported that RIP-V and RIP-L efficiently inhibit injury infection in a MRSA sepsis mouse model and increase the survival rate. However, due to rapid renal clearance and degradation, the plasma half-life of RIP and its derivatives *in vivo* is generally short, which greatly limits their clinical utility (Werle and Bernkop-Schnurch, 2006). RIP derivatives have been modified using amino acid substitution and oligomerization to improve their metabolic stability and activity *in vivo* and *in vitro*. 16P-AC (CH3CO-YKPVTNF-ST-YKPVTNF-CONH2), a hexadecapeptide RIP oligomer with an amidated C-terminal and an acetylated N-terminal, shows greatly enhanced stability and activity *in vivo* and is a promising drug candidate for treatment of MRSA-related infections (Zhou et al., 2016).

### Other Methods

Strategies that directly antagonize toxins may help prevent and treat *S. aureus* infections by reducing toxin-binding affinities or by blocking the ability of toxins to elicit pro-inflammatory responses and cytolytic activities. Toxins encoded by the core genome, such as the PSMs, α-hemolysin, LukGH, and SElX, are essential virulence factors for pathogenic staphylococci and are attractive for vaccine development (Cheung and Otto, 2012). For example, PSMs can cause lysis of red and white blood cells, stimulate inflammatory responses, facilitate neutrophil lysis after phagocytosis, advance biofilm-related infection propagation, and are key virulence determinants (Li et al., 2014). Since the amino acid sequences of PSMs are highly conserved between *S. aureus* strains, they are good targets for therapeutic antibody development (Cheung and Otto, 2012). In addition, export of PSM peptides is controlled by the dedicated Pmt secretion system (Chatterjee et al., 2013). Approaches that target PSM secretion may efficiently disable these toxins and prevent host damage (Li et al., 2014). Antibodies against α-hemolysin confer protection against *S. aureus* infection in various animal models (Hua et al., 2014). Rouha et al. (2015) isolated a monoclonal antibody with high affinity and cross-reactivity toward α-hemolysin and 4 different bi-component leukocidins (HlgAB, HlgCB, LukED, and LukSF). The antibody provided high levels of protection in murine models of pneumonia and sepsis (Rouha et al., 2015). HuMAb-154, a human monoclonal antibody with high affinity for SEB, prolongs survival of SEB-challenged mice by neutralizing SEB-induced cytokines (Drozdowski et al., 2010).

### Remaining Problems

Although *agr* systems have been targeted successfully in animal models, some problems remain. Although small molecule inhibitors that block the activity of the *agr* system and prevent gene expression are of great interest, most of them specifically block only one or two *agr* types. Inhibitors that can block all four *S. aureus agr* types need to be developed (Sully et al., 2014; Daly et al., 2015). *Agr* cross-inhibition may offer a basis for broader targeting, but a particular *agr* AIP or AIP derivative always exhibits varying activity in different staphylococcal strains. In addition, the therapeutic situation is complicated by the fact that different staphylococcal strains can coexist in a patient (Gomes et al., 2005). RNA-III inhibiting peptide has poor metabolic stability and activity. Although RIP derivatives can be stabilized by amino acid modification and oligomerization, more development is necessary before they can find use in treatment of human staphylococcus-associated infection. Another challenge is that numerous virulence factors are encoded by mobile genetic elements (MGES). This is a serious problem for target-oriented drug development due to their diversity and their parallel transfer between strains (Wright et al., 2005b). Toxins encoded by the core genome also vary. For example, the expression patterns for PSMs differ among *Staphylococcus* species, making it difficult to develop antibodies or vaccines against PSMs (Peschel and Otto, 2013).

A final puzzle is that *agr*-defective strains or *agr^-^* variants are detected in many infections (Traber et al., 2008). Most of these have lost the ability to disseminate in tissues and are often associated with biofilm formation (Painter et al., 2014). That a significant fraction of strains lacking *agr* activity have been isolated from cases of bacteremia has led to further consideration of the role of *agr* in invasive staphylococcal infection (Painter et al., 2014). One possibility is that the density of bacteria in the bloodstream is too low to activate the *agr* system. However, transcription analysis indicates that RNAIII expression is still low in blood even at high densities (James et al., 2013). Some studies suggest that AgrA and/or AIP activities may be inhibited by serum reactive oxygen species (ROS) (Kavanaugh and Horswill, 2016). Moreover, apolipoprotein B (apoB) in serum may sequester AIP from interaction with the sensor kinase AgrC and contribute to quorum-sensing inhibition (James et al., 2013).

Strategies to antagonize the components of the *agr* system will ultimately contribute to therapy against *S. aureus*-associated infections. However, the problems described above will need to be addressed before highly effective quorum sensing blockers can be developed to treat diseases caused by *S. aureus*.

## Author Contributions

LT was mainly responsible for writing the manuscript. SiL was mainly responsible for literature collection and assisted in writing. BJ provided guidance in writing. XH and ShL provided guidance on the ideas and grammar for the manuscript.

## Conflict of Interest Statement

The authors declare that the research was conducted in the absence of any commercial or financial relationships that could be construed as a potential conflict of interest.
